# Immunological Features of Neuroblastoma Amplified Sequence Deficiency: Report of the First Case Identified Through Newborn Screening for Primary Immunodeficiency and Review of the Literature

**DOI:** 10.3389/fimmu.2019.01955

**Published:** 2019-08-27

**Authors:** Silvia Ricci, Lorenzo Lodi, Daniele Serranti, Marco Moroni, Gilda Belli, Giorgia Mancano, Andrea La Barbera, Giulia Forzano, Giusi Mangone, Giuseppe Indolfi, Chiara Azzari

**Affiliations:** ^1^Section of Pediatrics, Division of Immunology, Department of Health Sciences, Meyer Children's University Hospital, Florence, Italy; ^2^Pediatric and Liver Unit, Meyer Children's University Hospital, Florence, Italy; ^3^Neonatal Intensive Care Unit, Department of Pediatrics, Meyer Children's University Hospital, Florence, Italy; ^4^Medical Genetics Unit, Meyer Children's University Hospital, Florence, Italy

**Keywords:** KREC, newborn screening, immunodeficiency, NBAS, neuroblastoma amplified sequence deficiency, SOPH, ILFS2

## Abstract

This is the first case of NBAS disease detected by NBS for primary immunodeficiency. NBS with KRECs is revealing unknown potentialities detecting conditions that benefit from early recognition like NBAS deficiency. Immune phenotyping should be mandatory in patients with NBAS deficiency since they can exhibit severe immunodeficiency with hypogammaglobulinemia as the most frequent finding. Fever during infections is a known trigger of acute liver failure in this syndrome, so immune dysfunction, should never go unnoticed in NBAS deficiency in order to start adequate therapy and prophylaxis.

## Background

Neuroblastoma amplified sequence (NBAS) deficiency is a very rare disease characterized by an extremely broad spectrum of phenotypes (1). Clinical features range from isolated recurrent episodic liver failure, precipitated by intercurrent febrile illness, to multisystemic syndrome, which includes short stature, skeletal osteopenia and dysplasia, optic atrophy, immunological abnormalities, and autoimmune disorders (2). NBAS is a highly conserved gene encoding for a component of the syntaxin-18 complex, a component of an endoplasmic reticulum (ER) tethering complex involved in the Golgi–ER retrograde transport of vesicles (3). Thermal susceptibility of the syntaxin-18 complex is the basis of fever dependency of liver failure episodes and could be involved in the possible pathogenic mechanism of NBAS mutations.

In this paper, we report the first case of NBAS deficiency identified in the context of a newborn screening (NBS) program for primary immunodeficiencies (PIDs) with κ-deleting recombination excision circles (KRECs) quantification on dried blood spot. We also focus on the immunological spectrum of the disease with a review of immunological cases in the literature.

## Case Presentation

The patient is a male newborn of Pakistani origin, son of consanguineous parents (Figure S1—Supplementary Material section), born by scheduled cesarean section at 36 weeks of gestational age, with severe intrauterine growth restriction (−3DS) and blood flow abnormalities at a prenatal Doppler ultrasonography.

**Figure 1:**
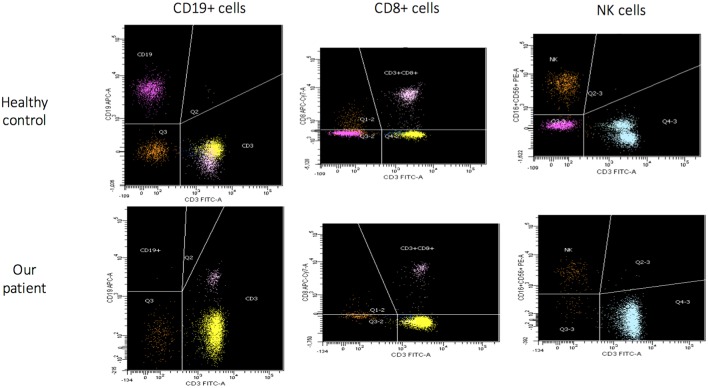
Flow cytometric assessment of lymphocyte subsets in healthy control and in our patient (age matched) that show for our patient absence of B cells (CD19+), low CD8+ and NK cells.

The patient came to our attention due to the complete absence of KRECs and normal TRECs on DBS (dried blood spot) while hospitalized for low weight at birth (1,520 g), intolerance for enteral feeding, hepatosplenomegaly, slightly elevated liver transaminase, head and face eczematous dermatitis, and persistent Rotavirus enteritis. During the 1st month, he also presented *Klebsiella pneumoniae* urinary tract infection and methicillin-resistant *Staphylococcus aureus* sepsis. Peculiar phenotypic features including triangular face, proptosis, flat philtrum, mild retrognatia, hirsutism, loose and slightly wrinkled skin, and apparent reduction of subcutaneous fat were noticed at birth. Complete blood count showed lymphocytopenia out of infectious episodes, marked hypereosinophilia, and a low platelet count with normal mean volume. Serum immunoglobulin G (IgG) were markedly decreased, IgA and IgM were undetectable, and levels of IgE were slightly augmented (Table 1). Extended phenotyping of the immune system was carried out on peripheral blood (Table 1, Figure 1) and confirmed on whole blood the normal expression of TRECs and the complete absence of KRECs, complete absence of CD19+ cells, low count of CD8+ lymphocytes, and reduced natural killer (NK) levels (Table 1, Figure 1). Classical and leaky forms of severe combined immunodeficiency were excluded by normal proliferation response of T cells to mitogens. Maternal engraftment of T lymphocytes was excluded by the normal representation of naïve T cells and by the different HLA-I typing and karyotypes of mother and son. The T cell receptor (TCR) repertoire expressed normal variability. Flow cytometric analysis showed normal expression of BTK (Bruton tyrosine kinase) protein on monocytes (Figure S2—Supplementary Material section) and normal expression of wasp protein on lymphocytes and monocytes. Regulatory T cells were normally represented among T CD4+ lymphocytes (Table 1). A colonoscopy was carried out for persistent diarrhea and reduced tolerance to enteral feeding. The histological examination of mucosal intestinal biopsies showed an absence of plasma cells and reduced representation of T lymphocytes, suggesting immunodeficiency but not autoimmune enteropathy. Molecular analysis for genome detection of adenovirus, rotavirus, EBV, CMV, and enterovirus were carried out on intestinal biopsies and no viral copies were detected. As a syndromic picture was suspected, clinical exome was performed by Next Generation Sequencing and identified a homozygous variant in NBAS (NM_015909): c. [1948C> T], p.Pro650Ser, inherited from both parents (Figure S1—Supplementary material section). This variant has not been described in the literature and is reported as rs558233705 with a low allele frequency in the Asian population in principal exome and genome databases (https://www.ncbi.nlm.nih.gov/snp/rs558233705#frequency_tab. Last visit on 05 February 2019).

**Table 1:** Patient's immune phenotyping at different ages.

**Age**	**1 month and 20 days**	**2-months**	**3-months**	**4-months**	**5-months**	**6-months**	**7-months**
**Lymphocyte count (cells/mcL) (% of WB)**							
Lymphocyte subtypes (cells/mcL) (% of lymphocytes)	2,813 (16,1%)	3,112 (18.5%)	714 (10%)	1,550 (21%)	1,835 (20%)	2,081 (15.1%)	638 (8.8%)
CD19+	4 (0%)	7 (0%)	3 (0%)	0 (0%)	0 (0%)	n.a.	n.a.
CD3+	2,348 (88%)	4,428 (95%)	1,963 (97%)	1,860 (95%)	1,575 (95%)	n.a.	n.a.
CD3+CD4+	2,127 (80%)	3,714 (79%)	1,396 (69%)	1,396 (72%)	1,370 (83%)	n.a.	n.a.
CD3+CD8+	189 (7%)	566 (12%)	389 (19%)	401 (21%)	186 (11%)	n.a.	n.a.
CD3−CD16+CD56+	261 (10%)	193 (4%)	43 (2%)	72 (4%)	60 (4%)	n.a.	n.a.
CD4+/CD8+ ratio	11.4	6.6	3.6	3.4	7.5	n.a.	n.a.
**Specific lymphocyte counts (% of CD4+)**							
CD45RA+	78%	n.a.	n.a.	n.a.	n.a.	n.a.	n.a.
CD45RO+	22%	n.a.	n.a.	n.a.	n.a.	n.a.	n.a.
CD25+CD127^Low^	n.a.	6.7 (n.r. 4–16)	n.a.	n.a.	n.a.	n.a.	n.a.
**Mitogen stimulation**							
PHA	85 (n.v. > 80)	n.a.	n.a.	n.a.	n.a.	n.a.	n.a.
IL-2	83 (n.v. > 80)	n.a.	n.a.	n.a.	n.a.	n.a.	n.a.
**Serum immunoglobulins**							
IgG (mg/dl)	325	213	n.a.	182	252	307	539
IgA (mg/dl)	<7.83	<7.83	n.a.	<7.83	<7.83	<7.83	8.2
IgM (mg/dl)	24	34	n.a.	<52.5	35	32	18
IgE (kU/L)	108	11	n.a.	n.a.	n.a.	n.a.	n.a.

*n.a., not available; n.v., normal values*.

To investigate the NBAS mutation-based disease features associated, total body X-ray was performed and revealed slightly bilateral brachydactilia of the 5th finger.

Immunoglobulin substitutive therapy and antimicrobial prophylaxis were promptly started during the 1st days of life. At the age of 3 months, due to the persistence of clinical manifestations with severe growth restriction and severe eczematous dermatitis, corticosteroid therapy was introduced with rapid improvement of gastrointestinal and cutaneous manifestations and the almost immediate reduction of peripheral hypereosinophilia. Steroid tapering was attempted unsuccessfully and caused re-exacerbation of gastrointestinal symptoms which, at 7 months of age, still constitute the most consistent clinical finding, together with severe growth restriction.

## Materials and Methods

Written informed consent has been obtained from the legal representatives of the patient for the publication of this case report.

## Newborn Screening for Primary Immunodeficiency With KRECs

The forward, reverse primers and probes for KRECs were designed in our laboratory using Primer Express software version 3.0 (Applied Biosystems). Primer specificity was assessed by BLAST search (http://www.ncbi.nlm.nih.gov/blast/), which confirmed their uniqueness. TREC and KREC levels were normalized per microliter of blood, assuming that the sample contains approximately 3 μl of whole blood. Calibration curves were generated by means of 10-fold serial dilution of plasmids that contain TREC, KREC, and β-actin sequences. Diagnostic cutoff scores were established at 25 TRECs/μl and 10 KRECs/μl, according to the values reported in literature (4).

## Whole-Exome Sequencing

Exomes of the patient and his parents were captured from the genomic DNA using SeqCap EZ MedExome (Roche NimbleGen) and sequencing was carried out using the NextSeq Illumina platform. The alignment of the reads (BWA), the call of the variants (GATK), the annotation (Annovar), and the prioritization of the variants were performed with strategies developed in-house according to the guidelines of the American College of Medical Genetics and Genomics (ACMG). The data obtained were filtered according to the patient's clinical phenotype. A homozygous variant in NBAS (NM_015909): c.1948C > T, inherited from both parents, was identified. The 1948C > T variant leads to the substitution of hydrophobic proline 650 with polar serine (p.Pro650Ser).

## Discussion

This is the first reported case of the NBAS mutation-based disease detected by NBS for PIDs with KRECs quantification on DBS. Bi-allelic mutations in the NBAS gene cause a wide range of phenotypes, including hepatic, skeletal, ocular, and immunologic abnormalities. T-cell receptor excision circles (TRECs) and KRECs are used worldwide for detection of T or B cell lymphopenia in newborns. As previously described (5), Tuscany is the only region in Italy to have implemented an NBS for PIDs with TREC and KREC quantification on DBS through multiplex real-time PCR (polymerase chain reaction). By January 2019, 38,300 children had been screened. The implementation and use of NBS for PIDs with TREC and KREC in a large-scale population are revealing otherwise unknown potentialities of the method. Not only does it allow the identification of known cellular and humoral PIDs, but it also permits the detection of clinically severe congenital conditions that benefit from early recognition and that are variably associated with immunodeficiency, such as NBAS deficiency. NBAS mutation-based disease has been associated with two main clinical phenotypes: the infantile liver failure syndrome 2 (ILFS2) and short stature with optic atrophy and Pelger–Huët anomaly syndrome (SOPH) (2, 6). The liver phenotype in NBAS deficiency is most typically a recurrent acute liver failure, triggered by febrile infections. The molecular pathogenesis by which an NBAS defect contributes to fever-dependent liver disease is not fully understood. The NBAS protein is thought to be involved in Golgi-to-ER retrograde vesicular trafficking and to control non-sense-mediated mRNA decay (7). A knockdown of NBAS in HeLa cells led to a defective protein glycosylation (3). Staufner et al. described how fibroblasts of patients with bi-allelic NBAS mutations showed an increased susceptibility to high temperatures at the protein and functional levels, causing an impaired transport between Golgi-to-ER retrograde vesicular trafficking. They suggested that raised temperatures themselves may be the starting point of ER stress-induced liver cell apoptosis that lead to fever-dependent ALF (acute liver failure) (1).

**Figure 2:**
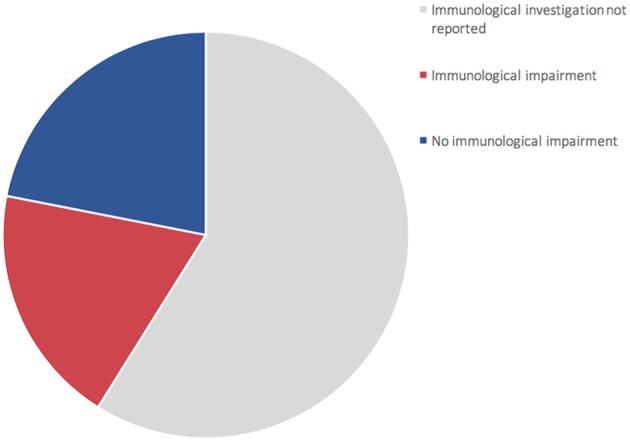
Representation of 73 patients reported in literature (1, 6, 8–22). 60% of patients (gray area) did not undergo complete immune characterization. 44% of patients who underwent immune characterization presented an immunological impairment (red area).

**Figure 3:**
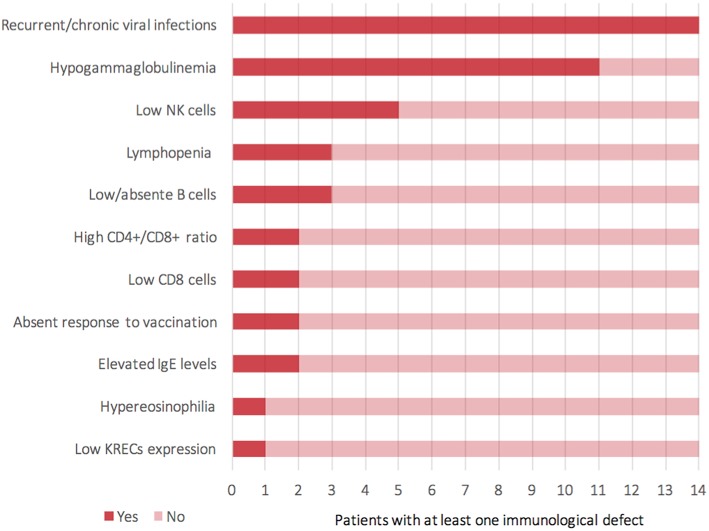
Representation of clinical and laboratory immunological findings (Y-axis) reported among 14 patients (X-axis) who presented a known immunological impairment (including the current case).

For the time being, our patient has not presented episodes of ALF, but persistent elevations of transaminase have been detected (Figure S3—Supplementary material section) with normal cholestasis values and coagulation function. The last abdominal ultrasound evaluation (performed during his 7th month) revealed liver of fairly increased size associated with preserved biliary tract features and liver echostructure. He presents facial dimorphisms and slight bilateral brachydactyly of the 5th finger, but no optical atrophy and no Pelger–Huët anomalies have been identified. Due to his young age, it is still difficult to determine whether the clinical phenotype tends toward ILFS2 or to SOPH. As in two previously described cases (8), the immunological impairment, associated with the gastrointestinal dysfunction, seems to be predominant in the global clinical picture of our patient. The literature reviewed revealed that, in more than half of the patients with NBAS deficiency, immune characterization is completely unknown (Figure 2) or partial (1, 6, 8–22). In particular, a complete absence of B cells has never been described in NBAS deficiency, but recurrent/chronic viral infections, severe hypogammaglobinemia, and a reduced number of B cells are the most frequently described immune anomalies in patients with NBAS deficiency (Figure 3) (1, 6, 8–22). Moreover, a reduced number of normally functional NK cells, altered CD4+/CD8+ ratio with reduced CD8+ numbers and hyper-IgE, have been previously described and are present in our patient (Figure 3). Our patient seems to possess the whole range of immune alterations hitherto described in previous cases, thus exhibiting the most severe immune phenotype ever reported in NBAS deficiency.

Immunodeficiency, associated with the NBAS phenotype, contributes to recurrent infections and abnormal liver enzymes with fever. Furthermore, since fever is a known trigger of acute liver failure, immune dysfunction, even if mild, should never go unnoticed in NBAS deficiency. In particular, hypogammaglobinemia is the most frequent immunological feature associated with NBAS deficiency (Figure 3) (1, 6, 8–22); thus, these patients may benefit from replacement therapy with immunoglobulin in order to reduce the rate of infections and the consequent risk of acute liver failure. We suggest that extended immune phenotyping should be mandatory in all these patients in order to collect and exchange data regarding a disease whose characterization is still being unfolding. In fact, basing on previously reported NBAS cases in literature, it is not possible to define a clear genotype–phenotype correlation regarding immunological involvement in this syndrome (Figure S4) (1, 6, 8–22). The phenotype classification for PID outlined by the International Union of Immunological Societies (IUIS) already includes NBAS deficiency among inborn errors of immunity (23). If the high frequency of hypogammaglobinemia in NBAS deficiency is confirmed (Figure 2), the authors feel that inclusion among prevalent antibody deficiencies could be of even greater support for the clinicians facing such a challenging diagnostic process. The potential mechanism of NBAS mutations affecting the immune system is still unknown. Further in-depth functional studies on Golgi-to-ER retrograde vesicular trafficking in immune cells of affected patients are needed to establish the genotype–phenotype correlations and to reveal the pathogenesis of the wide immunological phenotypic spectrum of the NBAS disease.

## Ethics Statement

A specific approval by the local ethical committee was not required because all analyses included in this study had been performed as part of the routine clinical activity. All data have been anonymized. Written informed consent was obtained from the parents of the patient for the publication of this case report.

## Author Contributions

MM and GB evaluated the patient during his NICU hospitalization. Evaluation on hepatic and immunological aspects was carried out by DS and SR, respectively. GManc and AL carried out the molecular diagnosis. GF collected the data for family pedigree. GMang performed the immunological tests. SR and LL collected the clinical and immunological data, reviewed the literature, wrote the first draft of the article, and critically revised it. GI and CA, as experts, critically revised the article and gave their final approval for submission. All authors read and approved the final manuscript.

### Conflict of Interest Statement

The authors declare that the research was conducted in the absence of any commercial or financial relationships that could be construed as a potential conflict of interest.

## Abbreviations

NBAS: neuroblastoma amplified sequence
TRECs: T-cell receptor excision circles
KRECs: κ-deleting recombination excision circles
SOPH: short stature with optic atrophy and Pelger–Huët anomaly syndrome
ILFS2: type 2 infantile hepatic impairment.
