# Supplementary figures and images for: Immunological Features of Neuroblastoma Amplified Sequence Deficiency: Report of the First Case Identified Through Newborn Screening for Primary Immunodeficiency and Review of the Literature
Source: Front Immunol. 2019 Aug 27;10:1955. doi: 10.3389/fimmu.2019.01955 (PMC6718460; doi:10.3389/fimmu.2019.01955)

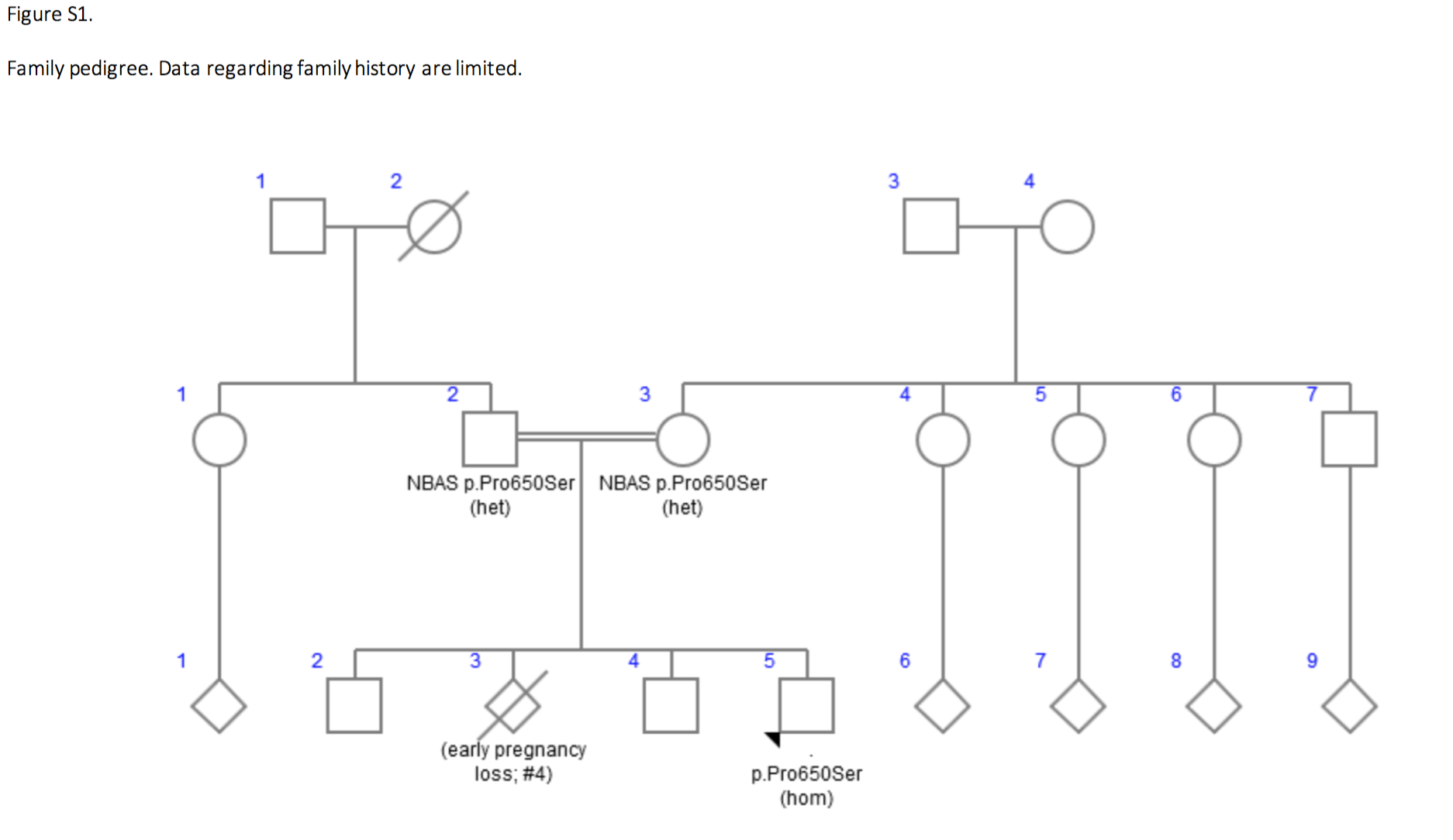

Supplement: Supplementary file 1 [file Image_1.TIFF]

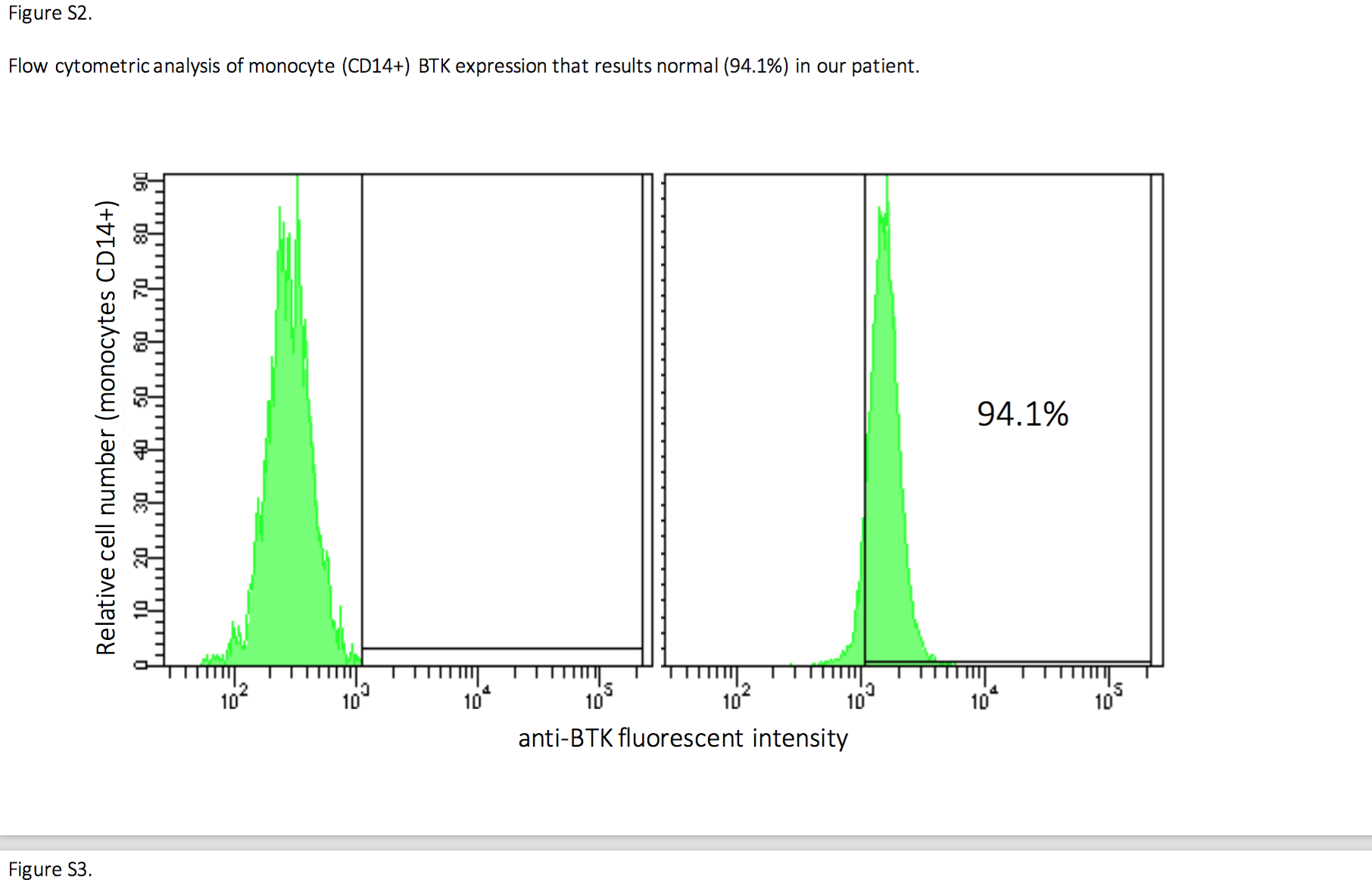

Supplement: Supplementary file 2 [file Image_2.TIFF]

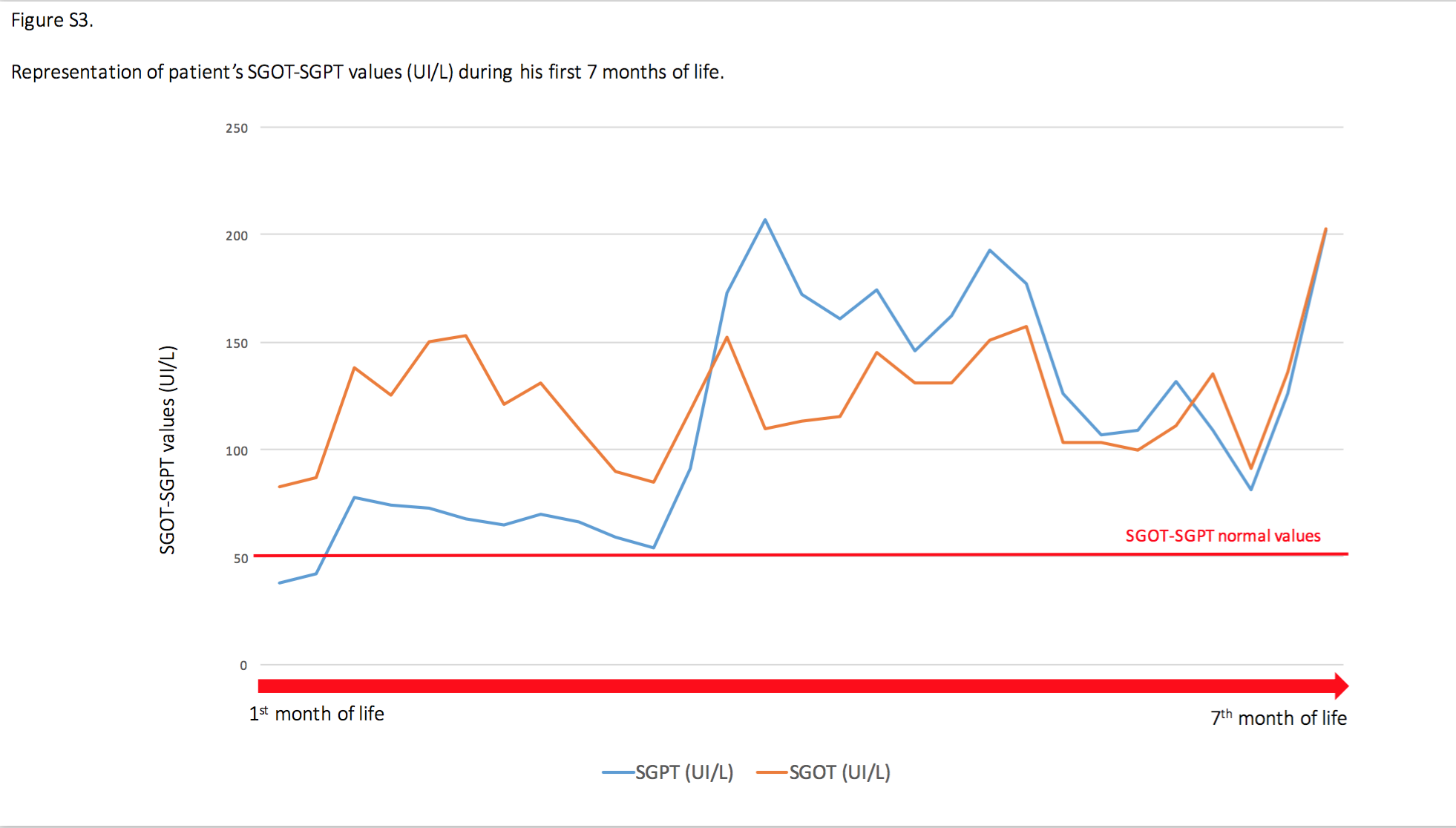

Supplement: Supplementary file 3 [file Image_3.TIFF]

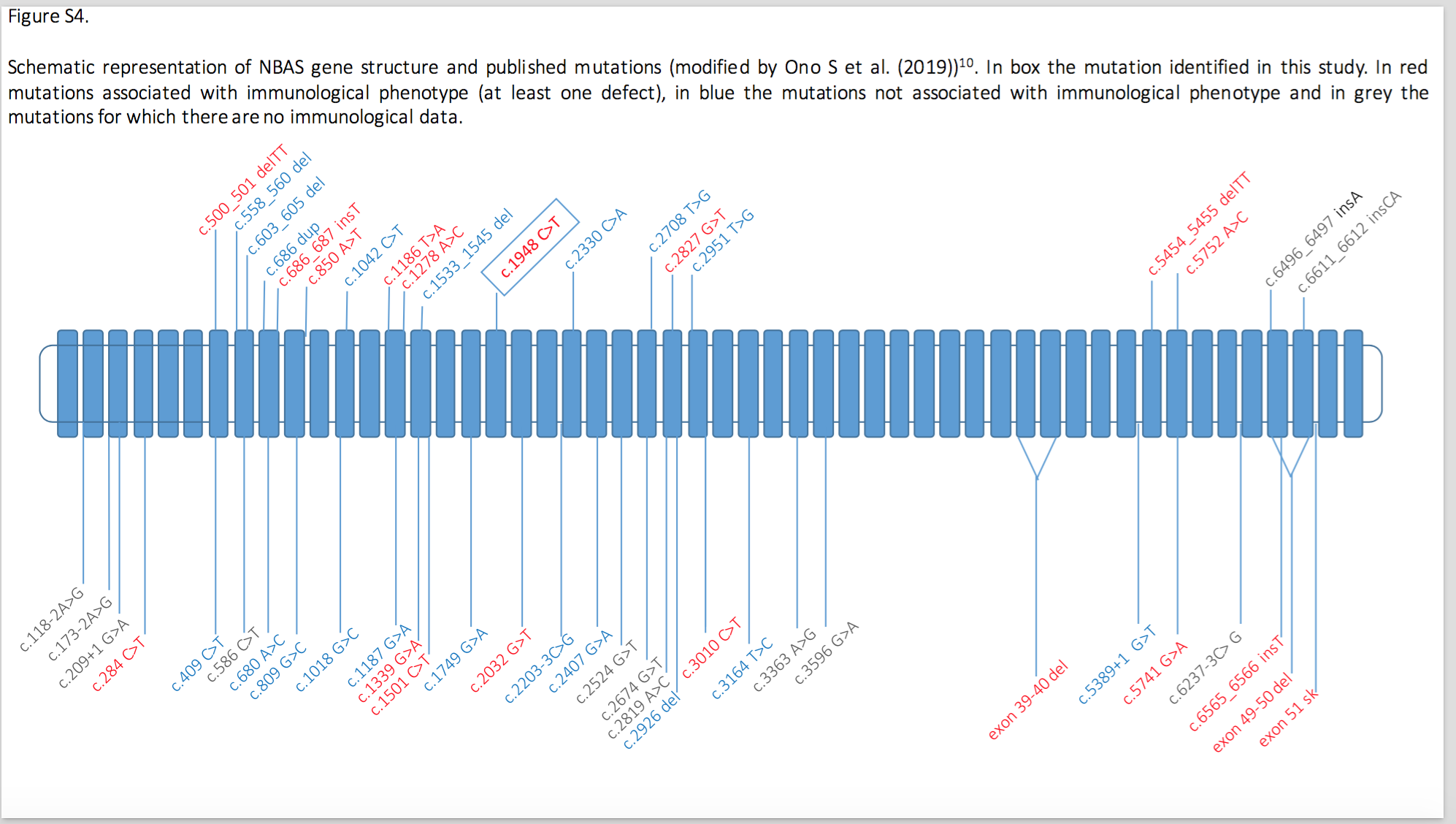

Supplement: Supplementary file 4 [file Image_4.TIFF]
